# Long-Term Follow-Up and Outcomes of Autoimmune Thyroiditis in Childhood

**DOI:** 10.3389/fendo.2020.00309

**Published:** 2020-06-05

**Authors:** Osnat Admoni, Shoshana Rath, Tal Almagor, Ghadir Elias-Assad, Yardena Tenenbaum-Rakover

**Affiliations:** ^1^Pediatric Endocrine Institute, Ha'Emek Medical Center, Afula, Israel; ^2^Rappaport Faculty of Medicine, Technion Institute of Technology, Haifa, Israel

**Keywords:** autoimmune thyroiditis (AIT), Hashimoto's thyroiditis, thyroid autoantibodies, goiter, Hashitoxicosis

## Abstract

**Background:** Autoimmune thyroiditis (AIT) is the most common cause of acquired hypothyroidism in children. The natural outcome of AIT in childhood has been reported previously however follow-up duration is generally short and results variable.

**Objectives:** To characterize clinical and biochemical findings at presentation of AIT, evaluate long-term outcomes and assess which factors at presentation predict evolution over time.

**Study cohort:** 201 children under 18 years of age at presentation (82% female) were enrolled. Subjects were divided into five subgroups according to thyroid stimulating hormone (TSH) level at referral.

**Results:** Mean follow-up was 8.1 years (range 0–29 years). At presentation, 34% of patients had overt hypothyroidism, 32% subclinical hypothyroidism (SCH), 16% compensated hypothyroidism, 14% were euthyroid, and 3.7% had Hashitoxicosis. Children with overt hypothyroidism were younger (10.6 vs. 13.2 years) and had higher thyroid peroxidase antibody titers. At the time of the study, levothyroxine (LT_4_) therapy was required in 26% of children who were euthyroid at presentation, 56% of SCH patients, 83–84% of those with TSH above 10 mIU/L, and 57% of those with Hashitoxicosis. Over the years, 16% of children presenting with overt hypothyroidism stopped therapy. Free T_4_ at presentation was the only predictor of outcome over time.

**Conclusions:** Our findings suggest that only 26% children who were euthyroid at presentation developed hypothyroidism, whereas over 50% of those with SCH went on to require treatment. Of those presenting with overt hypothyroidism, 16% recovered with time. The only predictive parameter for LT_4_ therapy at the end of the study was free T_4_ levels at presentation. Long-term follow-up is required to determine ongoing therapy needs and screen for additional autoimmune diseases.

## Introduction

Autoimmune thyroiditis (AIT), also known as Hashimoto's thyroiditis, is the most common cause of acquired hypothyroidism in childhood, with a prevalence of 1 to 3%, peaking during adolescence (1–3). There is a female predominance, with a female-to-male ratio of 4–8:1 (2). AIT is characterized by thyroid destruction due to an autoimmune-mediated process, resulting in gradual thyroid failure with or without goiter. The diagnosis of AIT is suggested by the presence of anti-thyroid antibodies against peroxidase (TPOAb) and/or thyroglobulin (TGAb) and by a typical hypo-echogenic and heterogeneous ultrasound pattern. Children are commonly referred for endocrine evaluation due to thyroid enlargement or on the basis of abnormal thyroid function results discovered as part of a medical workup for variable complaints (often unrelated to thyroid dysfunction) or for positive family history of AIT. At the time of diagnosis, thyroid function in children may be variable ranging from euthyroidism (52.1%), to overt (22.2%) or subclinical hypothyroidism (SCH) (19.2%) or, more rarely to either subclinical or overt hyperthyroidism (6.5%) (4).

Predictive factors for progression from euthyroidism or SCH to overt hypothyroidism, or for recovery from hypothyroidism over time, have been investigated in some studies, with variable results (3–19). Some of the studies included a small number of patients (5, 7, 8, 12, 19) and were of short duration (9, 10, 13, 16). Moreover, the definition of SCH, as well as indications for levothyroxine (LT_4_) supplemental therapy, differ among authors (15) and include goiter shrinkage in some instances (20). de Vries et al. (18) reported that almost all of their patients required LT_4_ treatment either at referral or during follow-up. Conversely, other studies suggested favorable outcomes for patients who were euthyroid or had SCH at referral (6–9, 11, 16), whereas only 25–50% of those with initial thyroid dysfunction experienced normalization of thyroid function over time (1, 8, 9, 12–14, 17).

Predictive factors for the development of overt hypothyroidism included: elevated TGAb and the presence of goiter (6), as well as elevated TPOAb, elevated thyroid stimulating hormone (TSH) and the presence of celiac disease (16).

The objectives of this retrospective study were to evaluate the long-outcomes of AIT in children with variable thyroid function at presentation, to evaluate the prevalence of euthyroid and/or SCH patients developing overt hypothyroidism, and the prevalence of patients with overt hypothyroidism becoming euthyroid after long-term treatment, and to identify predictive factors of long-term outcomes.

## Subjects and Methods

### Study Cohort

We enrolled 201 subjects in the study, all under 18 years of age at diagnosis. They were followed up on a 6-monthly basis at Ha'Emek Medical Center or in outpatient clinics affiliated with the hospital. All patients had positive TGAb and/or TPOAb. Most subjects also had at least one of the following: abnormal thyroid function, enlarged thyroid gland, morphological changes on thyroid ultrasound. Patients were referred for evaluation by a pediatric endocrinologist due to one or more of the following: abnormal thyroid functions, elevated thyroid antibodies, presence of goiter and various complaints that were related to abnormal thyroid function. Patients with diabetes mellitus type 1 (DMT1) diagnosed prior to the diagnosis of AIT, and those with Down or Turner syndromes, were excluded from the study. LT_4_ supplemental therapy was initiated in overt hypothyroidism, when TSH levels were above 10 mIU/L, and in a few individuals with TSH <10 mIU/L for the purpose of goiter shrinkage. LT_4_ dose was adjusted to maintain TSH concentrations within a normal range. Data were collected retrospectively from computerized medical files and included: clinical findings and thyroid function at presentation, during follow-up and at the last visit; LT_4_ therapy initiation; additional autoimmune diseases and family history of autoimmune and thyroid diseases.

### Hormone Analysis

Serum TSH (normal range 0.4–4.2 mIU/L) and free thyroxine (FT_4_; normal range 10–20 pmol/L) concentrations were measured by direct automated chemiluminescent immunoradiometric assays using ADVIA Centaur (Bayer Corporation, Tarrytown, NY). TGAb and TPOAb were measured by direct automated chemiluminescent immunoradiometric assay using Immulite 2000 (Siemens, Llanberis, Gwynedd, UK). TGAb and TPOAb were considered positive above 35 U/mL.

### Subgroups

Patients were classified into five subgroups according to TSH level at presentation: (i) hyperthyroid (TSH <0.03 mIU/L and FT_4_ ≥ 20 pmol/L); (ii) euthyroid (TSH 0.4–4.2 mIU/L and FT_4_ 10–20 pmol/L); (iii) SCH (TSH 4.3–10 mIU/L); (iv) compensated hypothyroid (TSH 10.1–20 mIU/L with normal FT_4_); (v) hypothyroid (TSH > 20 mIU/L). Since our practice is to initiate LT_4_ therapy in patients with TSH above 10 mIU/L even if FT_4_ is within the normal range, we divided patients into two subgroups according to their TSH levels: those with TSH 4.3–10 mIU/L were defined as SCH while those with TSH above 10 mIU/L and FT_4_ above 10 pmol/L were defined as compensated SCH.

### Statistical Analysis

Statistical analyses were performed with SAS v9.4 statistical software package (Cary, NC). Study groups were compared by Kruskal–Wallis test (continuous variables) and Chi-square or Fisher's Exact Test (categorical variables). Pairwise comparisons were made using Wilcoxon two-sample tests and Chi-square or Fisher's exact test for continuous and categorical variables, respectively, using Bonferroni correction. Univariate analyses were performed for the study outcome: LT_4_ therapy at the end of the study follow-up. Stepwise logistic regression was used for multivariable analysis to establish independent predictors for LT_4_ therapy at the end of the follow-up period. Significance was set at *p* < 0.05.

This study was approved by the Ethics Committee of Ha'Emek Medical Center.

## Results

The study population consisted of 201 subjects (female-to-male ratio of 4.5:1) with mean age of 11.7 ± 3.4 years (range 2.25–17.75 years). Reasons for referral were goiter or thyroid dysfunction and/or increased thyroid autoantibodies identified during a work-up performed for various other complaints (hair loss, obesity, weight gain, short stature, pubertal delay, fatigue and headaches).

Mean TSH at presentation was 55 ± 145 mIU/L (range 0.02–1,225) and FT_4_ 12.7 ± 6.7 pmol/L (range 1.2–67). TSH at presentation, was undetectable in 3.7% of subjects, within the normal range in 14%, slightly increased in 32%, and elevated (>10 mIU/L) in 50%. FT_4_ was below the normal range in 29% of patients. TGAb was positive in 83% and TPOAb in 95% of cases. Thyroid gland enlargement was identified in 66% of patients by neck examination.

Fifty-five patients (28%) had an additional autoimmune disease diagnosed either before or after AIT diagnosis. Autoimmune comorbidities included celiac disease (15 patients), pernicious anemia (nine patients), rheumatic diseases (six patients), vitiligo (five patients), DMT1 (three patients), alopecia areata (three patients) and others (14 patients). Positive family history of thyroid diseases was reported in 80 patients (40%), including AIT, Graves' disease, papillary thyroid carcinoma, and multinodular goiter. Follow-up duration was 8.1 years (range 0–29).

The cohort was divided into five subgroups according to TSH level at diagnosis: hyperthyroid (seven patients), euthyroid (27 patients), SCH (60 patients), compensated SCH (30 patients), and hypothyroid (64 patients). Clinical and biochemical findings in the different subgroups are presented in Table 1. Subjects with overt hypothyroidism were younger than euthyroid subjects (*p* = 0.0011). As expected FT_4_ levels were significantly lower (*p* < 0.0001) in overtly hypothyroid children compared to those with SCH and euthyroid patients. TGAb levels at presentation did not differ between groups, but TPOAb values were higher in the overtly hypothyroid group than in the euthyroid group (*p* = 0.0004). Gender did not differ between groups.

**Table 1:** Comparison of clinical and biochemical parameters in the five subgroups.

**At diagnosis**	**Hyperthyroidism**	**Euthyroidism**	**SCH**	**Compensated SCH**	**Hypothyroidism**	***P*-value**
Hormonal status	TSH <0.03 mIU/L FT_4_ > 20 pmol/L	TSH 0.4–4.2 mIU/L FT_4_ 10–20 pmol/L	TSH 4.3–10 mIU/L	TSH 10.1–20 mIU/L	TSH > 20 mIU/L	
No. of patients	7 (3.7%)	27 (14.3%)	60 (32%)	30 (16%)	64 (34%)	
Gender (F:M) % male	6:1 (14%)	25:2 (8%)	43:17 (28%)	22:8 (27%)	57:7 (11%)	
**At presentation**						
Age (years)	13.8 ± 2.6 (8.2–16.2)	13.2 ± 3.0 (7.7–17.8)	11.5 ± 2.9 (5.2–17.5)	12.0 ± 3.8 (4.5–17.6)	10.6 ± 3.4 (2.3–17.6)	0.005
TSH (mIU/L)	0.03 ± 0.01 (0.02–0.04)	2.4 ± 1.0 (0.9–4.1)	7.1 ± 1.7 (4.4–9.8)	13.5 ± 3.0 (10.1–19.6)	152.1 ± 226 (21.7–1224.7)	<0.0001
FT_4_ (pmol/L)	34.5 ± 16.1 (19.6–67.0)	14.8 ± 2.2 (11.3–19.2)	14.1 ± 2.3 (9.4–19.8)	13.3 ± 1.7 (10.3–17.1)	7.8 ± 3.1 (1.2–14.2)	<0.0001
TPOAb (U/mL)	681 ± 448 (65–1,000)	397 ± 321 (26–1,000)	721 ± 1005 (10–5,730)	645 ± 397 (35–1,000)	1,195 ± 2,010 (11–13,372)	0.01
TGAb (U/mL)	357 ± 408 (30–1,136)	451 ± 723 (28–3,000)	485 ± 868 (20–3,000)	529 ± 1,092 (25–4,284)	876 ± 1,463 (30–6,500)	0.5
**A****t LT**_**4**_ **therapy initiation**						
Age (years)	17.4 ± 4.5 (12.5–23.3)	14.8 ± 3.9 (9.2–22.4)	13.5 ± 3.7 (6.0–21.8)	12.8 ± 4.5 (5.0–23.3)	10.6 ± 3.4 (2.3–17.8)	0.0001
Years between diagnosis and LT_4_ therapy	4.4 ± 3.6 (1.07–9.5)	2.7 ± 2.1 (0–8.1)	1.4 ± 2.2 (0–11.3)	0.27 ± 1.3 (0–12.4)	0.05 ± 0.271 (0–2.17)	<0.0001
TSH at LT_4_ initiation (mIU/L)	47.6 ± 69 (6.4–150.6)	36.0 ± 49.2 (0.9–132)	17.2 ± 28.0 (5.0–150.4)	21.9 ± 29.1 (5.5–150)	153.1 ± 2,283 (5.3–1,223)	<0.0001
FT_4_ at LT_4_ initiation (pmol/L)	10.4 ± 6.9 (6.9–14.4)	11.4 ± 4.3 (3.4–16.4)	13.2 ± 2.4 (8.8–19.8)	13.0 ± 2.7 (4.4–17.8)	7.7 ± 3.1 (1.2–13.7)	<0.0001
**Time of study**						
Age (years)	21.6 ± 6.7 (15–33)	19.5 ± 6.3 (8.1–31.1)	18.7 ± 5.5 (9.4–31.0)	20.0 ± 5.9 (9.3–35.0)	18.9 ± 5.8 (5.1–33.0)	0.76
Follow-up duration (years)	8.0 ± 6.5 (0.4–19)	6.4 ± 6.1 (0–20.8)	7.6 ± 6.1 (0–20.8)	8.7 ± 6.7 (0.1–29.4)	9.3 ± 5.5 (0.8–22.0)	0.11
TSH (mIU/L)	5.2 ± 4.3 (0.02–11.6)	3.4 ± 3.4 (0.36–18.6)	6.4 ± 3.4 (0.4–18.6)	15.1 ± 53.3 (0.3–296)	13.1 ± 31.8 (0.04–196)	0.15
FT_4_ (pmol/L)	14.9 ± 8.4 (12.3–28.4)	14.9 ± 9.9 (9.9–19.9)	14.9 ± 7.2 (9.9–19.9)	15.7 ± 6.4 (11.4–21.1)	14.9 ± 5.6 (2.2–28.4)	0.9
TGAb (U/mL)	460 ±599 (34–1,255)	411 ± 833 (30–3,000)	540 ± 980 (20–3,000)	1,655 ± 4,363 (39–15,907)	443 ± 565 (21–3,000)	0.58
TPOAb (U/mL)	515 ± 456 (76–1,000)	390 ± 291 (24–1,000)	692 ± 866 (11–1,000)	632 ± 380 (29–1,300)	552 ± 535 (10–3,000)	0.59
No of patients undergoing LT_4_ therapy at time of study (%)	4 (57%)	7 (26%)	33 (56%)	25 (83%)	53 (84%)	<0.0001

*TSH normal range 0.4–4.2 mIU/L*.

*FT_4_ normal range 10–20 pmol/L*.

*SCH, sub clinical hypothyroidism, TPOAb, TPO abs; TGAb, Thyroglobulin antibodies*.

At LT_4_ initiation, patients with overt hypothyroidism were significantly younger than those in the euthyroid, SCH, and hyperthyroid groups (*p* = 0.0016) and had higher TSH and lower FT_4_ levels.

During the follow-up period, LT_4_ therapy was initiated in 100% of subjects presenting with overt or compensated hypothyroidism; however, at the time of the study, 5 (16%) and 11 (17%) of those patients, respectively, no longer required treatment.

No differences in variables at presentation were found between patients who were off therapy compared to those who required therapy at the end of the follow-up period. As for the other groups, three patients (43%) presenting with hyperthyroidism, 27 patients (44%) with SCH and 20 (74%) of the euthyroid group did not receive treatment. Follow-up duration, age at last visit and thyroid autoantibodies at the time of the study did not differ among groups (Table 1).

A comparison of thyroid antibody levels in the whole cohort revealed lower levels at the study endpoint compared to levels at the time of referral. Univariate analysis revealed that patients who underwent therapy at presentation were younger (11.4 vs. 12.7 years, *p* = 0.036), had higher TSH (73 vs. 5.0 mIU/L, *p* < 0.0001), lower FT_4_ (11.7 vs. 15.8 pmol/L, *p* < 0.0001) and higher TPOAb (920 vs. 521 U/mL, *p* = 0.002). Gender and TGAb did not differ.

At the end of the study, 122 patients were receiving LT_4_ therapy, while 64 were not. Looking for factors at presentation predictive of LT_4_ therapy at the end of the follow-up period, multivariable logistic regression performed for age, gender, TSH, FT_4_, TPOAb, TGAb, goiter, familial history of thyroid disease and additional autoimmune diseases revealed that only FT_4_ level at presentation was predictive for a future need for LT_4_ therapy.

## Discussion

In this study, we examined clinical and laboratory characteristics at presentation of AIT in children and adolescents and its natural progression. We found that about 75% of children euthyroid at diagnosis remained in this stable condition over time, whereas only 16% of those presenting with overt hypothyroidism attained remission.

The primary cause for referral to our service was thyroid function abnormality or elevated autoantibodies (68%) and the most prevalent complaint was thyroid enlargement (32%). Interestingly, goiter was identified in 66% of the patients on physical examination at presentation, in keeping with the high prevalence of goiter reported in previous studies (10, 11).

The prevalence of AIT is about 1.2 to 3%, with 87% of cases asymptomatic at presentation and spontaneous resolution occurring in 50% (1, 2). The benign evolution of euthyroid AIT in children has been reported in several studies (7–9, 12–14). Our finding, that only 26% of children who were euthyroid at presentation developed hypothyroidism during the follow-up period is consistent with these data, suggesting that most euthyroid children remain disease free over time. On the other hand, in the study of de Vries et al. (18) almost all patients were treated with LT_4_ at referral or during follow-up, however, their data suggested that appropriately monitored LT_4_ therapy, even in euthyroid children, does not appear to be harmful.

SCH is characterized by serum TSH levels between 4.2 and 10 mIU/L with normal FT_4_ levels. In the presence of thyroid autoantibodies, the diagnosis of AIT is most likely (20). Lazar et al. (21) demonstrated spontaneous normalization of TSH values in most patients with SCH without supplemental therapy. Female gender and initial TSH above 7 mIU/L were predictors of sustained highly elevated TSH. In this study however, the cohort was not limited to subjects with AIT. The risk of deterioration of thyroid status over time is higher in AIT compared to idiopathic SCH (53 vs. 11%) (5, 14, 15), as observed in our study, where 56% of those with SCH required treatment over time.

The presence of additional autoimmune diseases (6, 15, 16) and of genetic syndrome such as Turner and Down syndromes (14, 15) has been shown to increase the risk of deterioration in thyroid function. In our study, therefore, patients with these syndromes were excluded, as were children with DMT1 in whom thyroid seropositive antibodies were detected after the diagnosis of diabetes mellitus, as the natural history of AIT in these children differs (22).

Amongst our cohort, a small number of patients were treated with LT_4_ therapy for goiter shrinkage despite TSH levels below 10 mIU/L, an approach that is suggested in a few studies (23, 24). Even though LT_4_ therapy has been shown to reduce thyroid autoantibodies in patients with SCH and overt hypothyroidism, its beneficial effect on the evolution of thyroid function (11, 25), and on metabolic (26) and neurocognitive outcome (27–29) remains uncertain. In the United States, the prevalence of positive thyroid antibodies, in the disease-free population above 12 years of age, is about 10%, it is more prevalent in females and increases with age (30). TPOAb are significantly associated with thyroid function but TGAb are not (30). In our study, elevated TPOAb were associated with initiation of LT_4_ at presentation, but were not predictive for long-term LT_4_ treatment, whereas, in our multivariate analysis, only FT_4_ values at presentation predicted future treatment requirements in children with AIT.

Normalization of thyroid function over time has been reported to occur in 30–50% of children presenting with overt hypothyroidism (8, 9). In our study, supplemental therapy was initiated in all such patients with remission occurring in only 16% by the end of the follow-up period. Although this remission rate is lower than that reported in other studies, it suggests that reassessment of thyroid status is important given that 16% of those with overt autoimmune hypothyroidism may not require lifelong LT_4_ therapy.

Hyperthyroidism was the presenting symptom in seven patients (3.7%), with four developing hypothyroidism over time. Although hyperthyroidism is an uncommon presentation of AIT, awareness of this condition is important and should be differentiated from Graves' disease in order to avoid unnecessary suppressive therapy (3, 31).

A family history of thyroid disease was present in 40% of children with AIT, including Hashimoto's thyroiditis, Graves' disease, multinodular goiter and papillary thyroid carcinoma, suggesting a genetic susceptibility to thyroid disease. In addition, other autoimmune diseases were present in 28% of the cohort. These findings highlight the need for screening patients with AIT for additional autoimmune diseases.

This large, long-term retrospective study adds further information to the controversial issue of the natural history of AIT in childhood. The major limit of this study is its retrospective nature, with variable duration of follow-up among individuals.

In conclusion, this study indicates that 26% of children with normal thyroid function at presentation develop overt hypothyroidism, over 50% of those with initial SCH may eventually require LT_4_ treatment and 16% of those presenting with overt hypothyroidism may recover over time. FT_4_ at presentation appears to be the only factor predicting evolution of thyroid status. Further long-term studies on large cohorts of patients are needed to better clarify the natural history of AIT and identify prognostic factors for therapeutic intervention.

## Data Availability Statement

The datasets generated for this study are available on request to the corresponding author.

## Ethics Statement

This study involving human participants was reviewed and approved by Ha'Emek Medical Center Helsinki Comittee. Written informed consent for participation was not provided by the participants' legal guardians/next of kin, nor was written consent required from the local ethics committee since the study was retrospective collecting data from medical files.

## Author Contributions

OA collected the data and reviewed and revised the manuscript. TA, SR, and GE-A reviewed and revised the manuscript. YT-R drafted the initial manuscript, designed the study, coordinated and collected the data, and revised the manuscript.

## Conflict of Interest

The authors declare that the research was conducted in the absence of any commercial or financial relationships that could be construed as a potential conflict of interest.
